# Rethinking children’s right to leisure during school holidays

**DOI:** 10.1080/11745398.2023.2250473

**Published:** 2023-09-05

**Authors:** Stephanie Chambers, Susie Smillie, Nicholas Watson

**Affiliations:** School of Social and Political Sciences, University of Glasgow, Glasgow, United Kingdom

**Keywords:** children’s rights, children and young people, participation, inequalities, school holidays

## Abstract

Leisure and health are human rights that apply to both children and adults. Leisure can enhance health and enable people to participate fully in leisure activities. One of children’s main opportunities for leisure is during school holidays. Little previous research has focused on this time in children’s lives. This paper presents a review of the literature surrounding school holidays, providing a critique of educational and public health approaches that focus narrowly on children’s future outcomes that may be associated with how they spend their time during these leisure periods. It argues that a more sociological understanding, rooted within child-centred approaches to leisure, provides the opportunity for children’s agency, participation and citizenship to be investigated more fully.

## Introduction

Leisure and health are human rights (United Nations Universal Declaration of Human Rights (UDHR)) (UN General Assembly 1948). These rights apply not only to adults, but also to children and young people. The United Nations Convention on the Rights of the Child (Unicef 1989) identifies the ‘right of the child to the enjoyment of the highest attainable standard of health’ (Article 24). For leisure, Article 31 of the convention recognizes:

the right of the child to rest and leisure, to engage in play and recreational activities appropriate to the age of the child … States Parties … shall encourage the provision of appropriate and equal opportunities for cultural, artistic, recreational and leisure activity.

Within the UK, these rights have been recognized within policy, though operationalized more around play than leisure. As areas of health, education, sport and the arts, and local government are devolved in the UK, each of the four nations has taken a slightly different approach in implementing policy in relation to this Article 31. The Scottish Government’s Play Strategy has highlighted the importance of play in itself and that it need not contribute towards an external goal or reward (Scottish Government 2013). The Welsh Government claimed to have drafted the world’s first play strategy in 2002 (Welsh Assembly 2002). Their strategy outlines the role of play in reducing leisure inequalities between children, with local authorities given a statutory duty to provide for play in their areas (Welsh Government 2014). The Northern Irish executive also has a play and leisure policy that prioritizes the importance of play in the lives of children and young people (Northern Ireland Executive 2009). Although a Play Strategy for England was developed (Department for Children 2008), its implementation was hindered through the 2008 financial crash and the subsequent cuts to government spending that followed it (Voce 2015). The latter example highlights the precarious position of children’s right to leisure when cuts to public spending are being made.

Beyond both health and leisure being fundamental human rights, they are further interconnected in a dynamic relationship. Health status can determine the possibilities through which individuals and groups might engage in leisure. Leisure activities can have both a positive and negative impact on physical and mental health (Stebbins 2021). Leisure scholars examining the interconnection between the two concepts have generally defined health holistically and beyond the bio-medical paradigm as being more than the absence of disease (World Health Organization 1986). When considered within a leisure studies context, health includes both physical, mental, social and emotional wellbeing. It is also defined as a resource through which individuals or groups can be enabled to participate in family life, education, work or leisure (Kleiber, Hutchinson, and Williams 2002; World Health Organization 1986).

Like health, leisure is a contested concept. It is generally understood as being unpaid non-work time, however, this inadequately defines leisure for people who spend substantial time in education, particularly children and young people. This issue has been addressed in the revised World Leisure Organization’s *Charter for Leisure* which includes adults and children in its scope (World Leisure Organization 2020). Some scholars have considered the extent to which leisure is having the freedom to choose activities, and as an area through which individuals (specifically adults) can assert greater control in their lives compared with work or family responsibilities (Blackshaw 2010; Carr 2017; Neulinger 1974; Stebbins 2020). Nevertheless, there is recognition amongst leisure scholars that the social determinants of both health and leisure overlap, and control in relation to both is severely restricted by socioeconomic circumstances and exclusionary social structures (Peel, Maxwell, and McGrath 2021). There is also controversy over the extent to which leisure must be valuable, either in terms of personal growth or production of a societal good (Dumazedier 1974; Kalimtzis 2017; Rojek 2010; Veal 2019). This inevitably raises the question of how value is measured and whose value is considered most important, and this is even more complicated when the value of children’s leisure is being discussed.

In considering children’s leisure, it is important to also consider the role of play. Play and leisure are interrelated concepts in that they can both be experienced outside of education or work time, although with the turn to play-based learning in education, this distinction is less clear than it once was. Within the context of this paper, play is understood to be pursued as an integral part of many leisure activities, and particularly so in the case of children’s leisure (Stebbins 2015). Both leisure and play have become instrumentalised as a way of supporting children’s health and education rather than as activities in and of themselves. Play, for example, has become medicalised and is seen as a way of tackling obesity or of increasing children’s physical activity. Alexander, Frohlich, and Fusco (2014, 158) describe play as having been ‘re-shaped as a purpose-oriented activity to promote children’s health’ rather than something that is purely pleasurable or enjoyable. There is a danger that a sole focus on health ignores the pleasure and enjoyment children and young people may seek from play, privileging those activities that are deemed as health enhancing and ignoring activities that may help to foster better social and emotional wellbeing (Alexander, Frohlich, and Fusco 2014). By focusing on children’s leisure, rather than solely play, there are opportunities to consider a wider range of activities that are important to children and young people. Mukherjee (2020) for example has categorized children’s leisure as structured, family, and casual – a much wider conceptualization than play.

Although there are few theories outlining the connection between leisure and health, some key themes include the advantages that arise when people are kept busy, enjoyment and relaxation, personal growth, identity formation/affirmation and as a coping strategy. This paper will highlight the extent to which these themes are absent in much of the literature surrounding children’s leisure time during school holidays. The mechanisms through which leisure is theorized to impact health is through the increase or decrease in risk associated with health promoting or damaging practices (Mansfield 2021), and this is the main focus of existing literature. Leisure activities associated with health are wide ranging but include physical activity, food work, creative and cultural activities, reading and screentime. Leisure studies scholars have challenged this risk management focus, arguing that it fails to account for the wider social determinants that impact on health and wellbeing (Peel, Maxwell, and McGrath 2021).

Within leisure and health studies little consideration has been given to children and young people’s (CYP) experiences of the connection between leisure and health. This paper builds from Mansfield’s (2021) call to progress understanding around leisure and health through a critical commentary of children’s leisure during school holidays. School holidays, particularly summer holidays, are the most substantial blocks of time that are assigned to children to engage in leisure activities. Summer holidays can range from 3 to 12 weeks in different settings. It is therefore surprising that in spite of these large blocks of leisure time assigned to CYP, there has been very little focus within leisure studies on how they spend this time, and the implications of this for CYP. Indeed, there has been relatively limited academic focus, particularly a critical focus, on CYP’s leisure time more generally. This is only now being readdressed. Mukherjee (2020) called for a ‘sustained dialogue’ between leisure and childhood studies which could allow for a critical examination of CYP’s leisure participation within the context of agency, social justice and lived citizenship.

We wish to engage in this dialogue through a consideration of school holiday programmes, particularly those free-to-access, in enabling children to realize their rights to both leisure and health. School holidays and their potential to impact CYP’s health and wellbeing positively or negatively have been considered from educational or public health approaches. After a brief outline of the history of school holidays and the UK policy context surrounding them, this literature will be outlined and the future-oriented nature of the outcomes measured within them, and their lack of consideration of sociocultural context, critiqued. We also acknowledge some of the advantages of these disciplinary approaches, as well as challenging those researching holiday programmes to broaden their understanding sociologically. The paper will argue that a more sociological approach can offer greater insight into structural inequalities in access to programmes, and the opportunities within them to provide CYPs opportunities to develop their citizenship, agency and voice. The benefits of approaching school holidays with an interdisciplinary lens are that the ambiguities, complexities, and tensions of CYP’s lives during summer holidays can be investigated. Some of these tensions include the need to protect the vulnerable, whilst not being overly prescriptive; trade-offs between structured and unstructured activities; pursuing social justice and redistribution of opportunity versus surveillance and policing of activities.

## A brief history of school holidays

School holidays can conjure idealistic notions of long summers of children playing happily outdoors. With that in mind, it is not a major leap to believe that school holidays are scheduled to provide opportunities for CYP to engage in leisure, including essential rest, relaxation and enriching non-academic activities (Patall, Cooper, and Allen 2010). More has been written on the history of school holidays in the USA than in other countries, but there is a widespread perception that school calendars were related to agrarian calendars and the need for children to help parents to plant and harvest food on farms rather than to provide leisure opportunities (Glines 1995). Whilst this was the case in rural areas in the 19th and early 20th centuries in the USA, schools in urban areas were open for 11 or 12 months of the year (Silva 2007). This suggests that community-specific needs were more likely to determine the structure of the school year (Gold 2002). Standardization of the school calendar in the USA occurred over the twentieth century, as rural schools added more days and urban schools reduced days, with the summer break lasting up to 12 weeks in some areas. A number of reasons have been put forward for the move towards standardization, including increased urbanization. As more families moved, larger schools were built with the ability to stream pupils by grades, leading to the requirement for a specific school year start date. By having a longer break during the summer, families were provided with an opportunity to move and settle before schools began. Other explanations for the structuring of the calendar include the need to avoid intense heat in the height of summer, the fear of increased disease incidence in hot and humid conditions, wealthy families vacationing (Bloom 2009; Patall, Cooper, and Allen 2010) and financial limitations related to keeping schools open for more days during the year (Weiss and Brown 2003).

School summer holidays in England, Scotland and Wales are around six weeks in length and eight weeks in Northern Ireland. The right to leisure for CYP during this time has been recognized for more than 100 years. In 1902, Mary Tanner advocated for children living in difficult urban conditions in London to be afforded the same opportunities for leisure and enrichment as rural dwelling children (Tanner 1902). She emphasized the importance of granting children freedom and play opportunities, using the vast London parks as civic assets. She identified the inequalities arising from limited access to these parks as affecting children's leisure experiences. She expressed concern about children's wellbeing when their only option for play was unclean streets and advocated for vacation schools. Describing a visit to a vacation school in London, she highlighted the diverse enrichment activities available, such as drama, gymnastics, and storytelling, underlining the necessity for schools to enable children to connect with nature. However, it was evident that her perspective also aimed to control working-class children through a ‘civilising’ endeavour:

The life of a working-class child with its sordid preoccupations is not calculated to cultivate its imagination … to counteract this atrophy of the imaginative faculty, which is far more socially mischievous than may appear at first sight, should be the primary aim informing the whole curriculum of the holiday school. (326)

The tensions highlighted by Tanner in terms of promoting participation, equality and health versus ensuring working-class children are suitably occupied during school holidays are still relevant today and will be returned to throughout this paper.

## School holidays, learning and wellbeing losses, and socioeconomic inequalities

Critics of the prevailing structure of school calendars have argued that the rationales provided in the past for a long summer break no longer apply and are detrimental to CYP. They focus on CYP’s right to education and health, with leisure problematized unless it is contributing towards attainment of some kind. Potential deficits in academic performance have been identified after long holidays, particularly in the US, and described as ‘summer learning loss’. A seminal US study reported that much of the inequality in educational attainment between those from more and less deprived areas resulted from differences emerging over the summer holidays (Entwisle and Alexander 1992). This was reinforced by Cooper et al.’s (1996) systematic review and meta-analysis of summer learning loss. The main findings from included studies were that there was a loss in ‘learning’, including reading, spelling, and math computation and concepts. In addition, for reading, inequalities were identified with children categorized as middle-class gaining in reading ability on their return to school after the summer, and children categorized as lower-class reporting lower reading ability on their return to school after summer.

More recently, research in this area has produced mixed results and has indicated that much of the previous identified differences were the result of methodological design, including a focus on only the youngest children, and measures with poor reliability and validity (Kromydas et al. 2022). Indeed, inequalities in educational attainment due to differential summer holiday experiences are less than previously thought (Kromydas et al. 2022; von Hippel and Workman 2016). Kuhfeld (2019) has rejected the term ‘learning loss’ and has argued that all children experience a ‘slow-down’ over summer. They highlight that there is also very little evidence on whether programmes to mitigate this loss are successful.

There has also been some focus on health and wellbeing losses after school holidays, including differences in measures of physical health and health behaviours including diet, physical activity, sedentary behaviour, screen time, sleep (Brazendale et al. 2017; Brazendale et al. 2018; Carrel et al. 2007; Nagy et al. 2019; Staiano, Broyles, and Katzmarzyk 2015; von Hippel and Workman 2016; Warner, Murray, and Meyer 2008; Zinkel et al. 2013), physical safety (Tran, Holland, and Bertinetti 2021), and social and emotional wellbeing (Morgan et al. 2019). Kromydas et al. (2022) reported that CYP’s social and emotional wellbeing worsened over summer holidays for 7- and 14-year-olds, and that inequalities were larger after holidays at these ages too. Morgan et al. (2019) surveyed over 100,000 children in Wales, reporting an association between negative experiences during summer holidays (hunger, loneliness, lack of time with friends and physical inactivity) and poorer mental health outcomes on return to school. A structural equation model found that the relationship between mental wellbeing and family affluence was mediated by children’s summer holiday experiences. Findings from before/after school holiday studies on wellbeing losses must be treated cautiously as they may not be capturing the effects of a summer holiday, but instead be measuring the response to restarting school.

The research outlined in this section highlights potential areas of concern in children’s learning and wellbeing outcomes. Nevertheless, this research does not take a child-centred, or children’s rights perspective by considering the wider benefits of leisure in CYP’s lives (James, Jenks, and Prout 1998). Outcomes are measured through standardized testing, or large-scale surveys, with those outcomes designated as important based on adult-researcher and policymaker agendas. The importance is generally related to children’s future citizenship status, as either contributing economically to the state through employment, or avoiding burdening the state through accessing healthcare. There is little focus on children’s role as active agents and their experiences of both school and holiday time and the other benefits they may enjoy during any break from school. In addition, CYP are rarely asked if they have enjoyed school holidays. Patall, Cooper, and Allen's (2010) review is one of the few that recognizes that school holidays can be opportunities for other forms of learning and for leisure. There is some evidence that parents recognize the non-educational opportunities that summer provides, including personal rest and relaxation through family connection (Olsen, Garst, and Powell 2019). Overall, however, there is comparatively little focus on how addressing any losses in learning or wellbeing might negatively impact on children’s right to leisure.

## School holidays, inequalities and policy responses

School holiday inequalities have received greater policy focus in the UK since around 2010. The global financial crisis in 2008, and the subsequent austerity measures which followed it, led to a focus on how families could meet basic needs, the role of the state, and the potentially devastating long term impacts of child poverty and family food insecurity. The specific cost of school holidays to families has received considerable attention by advocates. Childcare costs during school holidays are high and are estimated to cost families across the UK on average £148 per week (Broomé, Kunwar Deer, and Shorto 2022). Private and voluntary sector provision costs on average 21% more than public sector provision. There are substantial differences in the availability of public sector provision across the UK, with 26% of provision delivered via public sector providers in Scotland, compared with only 3% in Wales (Broomé, Kunwar Deer, and Shorto 2022). The Child Poverty Action Group commissioned a literature review highlighting the cost of food, day trips and other holidays (Stewart, Watson, and Campbell 2018). The report concluded that children from areas of high deprivation were less likely to engage in activities during the summer and that childcare was not only expensive, but that its availability was limited. Before the COVID-19 pandemic, wellbeing concerns were more concentrated on access to holiday camps and programmes, with the recognized inequality that parents on higher incomes could pay for high quality, enriching activities for their children. Those on lower incomes did not have access to these opportunities, and the lack of affordable provision impacted on parents’ ability to work, increasing the likelihood that families experienced poverty. Enforced school closures during the pandemic led to greater media and policy attention on what happens to CYP when they are not in school, and the extent to which absence of enriching activities might impact on their education, health and wellbeing (Hefferon et al. 2020; Larsen, Helland, and Holt 2022; Maftei, Merlici, and Roca 2022; Melegari et al. 2021; Naff et al. 2022; Prime, Wade, and Browne 2020; Stassart, Wagener, and Etienne 2021; The Children's Society 2020). Questions were raised particularly in relation to children and young people’s social and emotional wellbeing. There has also been considerable concern around the rise in obesity rates since the beginning of the COVID-19 pandemic, with lack of physical activity during that time identified as a likely contributing factor for this trend (World Health Organization 2022).

Food insecurity during school holidays has dominated much of the UK policy agenda in this area. Many UK children receive a free school meal each day they attend, with universal systems in place for younger children and means testing for older children based on family income and welfare support status. With austerity measures, advocacy groups and some politicians began to question how families qualifying for this entitlement provided meals during school holidays when a free meal was unavailable (Stewart, Watson, and Campbell 2018). During the COVID-19 pandemic this issue was raised again as food provisions for families were exposed as being inadequate, driven by campaigning by England footballer Marcus Rashford (Sinha et al. 2020). His campaigning, along with advocacy groups, forced the UK Government to reverse their original decision to end the free school meal funding during the pandemic restrictions once school holidays began.

The political focus on what happens when children are not in school has resulted in both local and national policy action to ensure that some out of school provision is in place. In some areas, families receive direct payments for food during holidays, but in others, various programmes run providing food and activities. The UK Government has committed to fund their Holiday Activities and Food Programme from 2022 to 2024. In 2021, the Scottish Government invested £20 million in a Summer of Play, with £10 million made available for 2022. The focus on play is interesting, and is in line with the Scottish Government’s Play Strategy (2013) and Education Scotland’s focus on play in education and active learning (Education Scotland 2022). In discussions of post-pandemic recovery, concerns had been raised about the length of time in which CYP were out of school, and the possibilities of catch-up classes running over summer holidays. In contrast, others resisted these calls, emphasizing children’s need for recovery through play and social connections (Weale 2021).

With these concerns around learning and health and wellbeing, particularly following COVID-19 school closures, there are now more opportunities available to engage children in structured programmes of activities during summer holidays. These opportunities are instrumentalised within UK public policy models of ‘healthism’ where activities, such as sport, are put forward as an unquestionable route through which to achieve behaviour change, rather than being framed as worthy leisure activities in their own right (Mansfield 2016). Many activities are available via payment by families whilst others are free to access, delivered through third or public sector organizations. Public health is focused on identifying the most effective ways through which to improve health and wellbeing through prevention activities, but also to reduce inequalities in health. At this time, there is limited evidence for the effectiveness of holiday programmes in improving CYP’s health and wellbeing. The evidence that does exist is limited in scope i.e. US-focused and small in scale (Hunt et al. 2019; Kidokoro et al. 2022; Moreno, Johnston, and Woehler 2013; Park and Lee 2015; Weaver et al. 2020). There is also substantial variation in programme content and delivery and often unclear programme aims/outcomes meaning that programme theory (the hypothesized causal mechanisms likely to result in positive CYP outcomes) is vague or non-existent. More needs to be understood in terms of a realist evaluation approach (Pawson, Tilley, and Tilley 1997) i.e. what works for whom and in what circumstances? There are opportunities therefore within public health approaches to summer holiday programmes to take a broader view of the processes within any programmes beyond measuring health-related outcomes via quantitative studies to fully understand their contexts, including fun and enjoyment.

Understanding the ways in which CYP’s right to leisure is realized, and the impact this has on them, undoubtedly requires an interdisciplinary approach. The literature discussed above is rooted in the disciplines of education and public health. These disciplines tend to be future-oriented. In many ways they take a lifecourse approach, that is understanding that experiences in childhood are likely to have a positive or negative impact through to adolescence, early, middle and older adulthood. While there is much to be gained from thinking about how the ways in which CYP spend their time now impacts on them in the future, there is a substantial likelihood that it fails to consider adequately CYP as active agents within society and their participation in leisure experiences. To achieve greater depth of understanding, much can be gained from drawing from leisure studies and a more sociological approach to the study of school holidays. Such approaches can provide a deeper consideration of contexts, and differences in the distribution of power and resources that determine the economic, material and psychosocial conditions in which children grow up.

## Childhood studies perspective

As discussed above, the tension between future-oriented perspectives on CYP’s holiday experiences and the consideration of their current experiences requires further examination. Childhood studies has extensively considered the dichotomy between children as beings versus becomings (Balen et al. 2006; James 2011; Prout 2011; Uprichard 2008). It recognizes that whilst structures constrain CYP, at the same time they have agency and voice (Giddens 1984). Childhood studies has sought to move away from a developmental focus in understanding CYP’s lives to a more sociological understanding (Woodhead 2009). Public health and education often perceive CYP as individuals in the process of becoming, needing protection and nurturing to ensure their future economic success and reduce the burden on health and social care systems. This can weaken children's status as citizens within neo-liberal systems, where individuals are responsible for managing their own health to avoid burdening the state (Bradford and McNamara 2015; Fullagar 2002; Peel, Maxwell, and McGrath 2021; Peterson and Lupton 1996). Such conceptualisations should be guarded against in the context of school holiday provision, as they risk co-opting children into narrow health frameworks. Embracing the perspective of children as beings and full citizens shifts the focus from the future to how they spend their time outside of school now, and whether the state provides equal opportunities for leisure participation. A broader understanding of the benefits of holiday provision can counteract an obsession with future health and wellbeing outcomes and emphasize the importance of connection, relationships, and enjoyment. Childhood studies also rejects the universality of a developmental approach to childhood, recognizing the importance of different contexts in lived experience (Burman 1994). Of course, there are nuances in all disciplines. Within public health, process evaluations of programmes often speak with CYP about their experiences and their recommendations for improving them (Cox, Campbell-Jack, and Blades 2022; Defeyter et al. 2018; Geary, Awoyemi, and Gracey 2022). There is some focus on enjoyment of the experiences and the specific activities, but that tends to be in the context of a programme theory or logic model where these are intermediate outcomes likely to lead to longer-term, and perceived to be, more important educational or health outcomes. Adopting a sociological leisure studies approach can provide deeper insights into CYP's present experiences, focusing on enjoyment and perceived connectedness (Mansfield, Daykin, and Kay 2020). This aligns with Mansfield’s (2021) call for a comprehensive understanding of the relationship between leisure and wellbeing by examining the underlying personal and societal processes and mechanisms connecting these two concepts.

Mansfield (2021) also flags the need for the policy implications of processes and mechanisms to be considered. When programmes become formalized, and public money is involved, tensions arise between adult versus child-centred approaches. Governments typically place conditions on organisations’ funding. For example, the UK Government states that the Holiday Activities and Food Programme should provide care for at least 4 h per day, at least one healthy meal per day, fun and enriching activities and improve nutrition knowledge (Cox, Campbell-Jack, and Blades 2022). Funding is always limited and what is offered is usually constrained by these very real practicalities. There is little evidence however that CYP are being consulted on what they would like to participate in when these policies and programmes are being developed and implemented.

A solely public health or educational approach fails to engage in a critical discussion of CYP’s agency, social positioning and lived citizenship. School holidays are framed as problematic times, particularly in terms of economic productivity. Parents lacking affordable childcare are unable to work (Statham, Parkes, and Nanda 2022), and are positioned as being an additional burden on the state in such circumstances, particularly single parents. There is limited discussion of the importance of a break from formalized learning, engagement in play or opportunities to experience boredom. CYP not attending paid-for-activities, or going on family holidays, are positioned as vulnerable and in need of protection. Within academic work on school holiday programmes, there is a deficit model of CYP’s citizenship with limited understanding of CYP’s lives and the role that free-to-access programmes might play in terms of participation. There has been no real attempt to understand how CYP spend time during the school holidays either in programmes or not in them. This limits understanding of the provision needed and what CYP might otherwise do when provision is unavailable. For those attending programmes, little is known about experienced power dynamics. Mukherjee (2020) has described children as political actors and discussed the micropolitics of child–child interaction in leisure spaces, but we must also consider child–adult interaction (Esser et al. 2016). There are also interactions to be considered between providers and funders, and where relevant, between funders and Government, as well as the role of the wider family. As with all policy interventions, these programmes reproduce power structures and therefore the power dynamics involved with them is of interest. Speaking with CYP about their experiences and opinions in this area provides opportunities for participation and voice.

In terms of children’s rights, public health-informed work on school holidays has highlighted the reproduction of inequalities in children’s leisure (Brazendale et al. 2017; Morgan et al. 2019; Park and Lee 2015). Austerity is recognized as a social determinant of health and its impact on child poverty and health and wellbeing outcomes has been well documented (Rajmil et al. 2018; Rajmil et al. 2020,). Public health acknowledges the inherent dangers that further implementation of neo-liberal frameworks presents (Viens 2019). Consumerism and private sector provision of leisure risks increasing inequalities, particularly in relation to physical activity and mental health (Bradford and McNamara 2015). Austerity has seen public sector leisure provision cut, whilst the voluntary sector has had to step in. Families on low incomes are less likely to be able to afford to pay the ‘market rate’ for activities (Schneider, Hastings, and LaBriola 2018). Public health approaches to school holidays recognize that some CYP are afforded the opportunity to learn and develop skills, make friends and to be cared for during this time, whilst others either spend time alone or in situations that could potentially harm their wellbeing (Morgan et al. 2019). In calling for state provision of food and activities, there is an attempt to enable families to access similar enrichment activities at no or low cost (Defeyter et al. 2022; Stretesky et al. 2020). There is therefore potential for school holiday programmes to be viewed as a means through which social justice can be pursued (Mann et al. 2018).

This paper, and literature in this area, has focused on socioeconomic inequalities, but other forms of inequality that have received little examination must be considered. Geographical, racial/cultural and disability-related inequalities, including the extent to which any programmes offered are inclusive and accessible, also need to be examined and the ways that these inequalities might intersect with socioeconomic inequalities (Mann et al. 2018). For families with non-white ethnic backgrounds, leisure can be a site through which parents seek to offer their children further opportunities to understand and experience their cultural heritage (Hallmon et al. 2021; Mukherjee and Barn 2021). It is unclear the extent to which holiday programmes offer these opportunities. For other parents from non-white backgrounds, leisure decisions are focused around opportunities to avoid racism and discrimination or to avoid tokenistic engagement with cultural differences (Hallmon et al. 2021; Mukherjee and Barn 2021). For CYP with physical disabilities, we know that leisure participation is more constrained than for CYP without a disability (Powrie et al. 2015). The likely impact of this lower participation is that CYP with disabilities have fewer opportunities to fulfil psychological needs, such as autonomy, relatedness and competence, as well as to experience the enjoyment that can come from leisure participation (Powrie et al. 2015). Evaluations of holiday programmes therefore need to consider the extent to which programmes reach those CYP most likely to benefit, offer equality of opportunity and meet the needs of the families engaging with them. Consideration also needs to be given as to whether there are differences in these outcomes between what might be available privately and free-to-access provision.

We must also consider our understanding of children’s leisure more generally. This is an area within leisure studies that is comparatively neglected, particularly for younger children. Children’s leisure is typically incorporated within family leisure. More is understood about play, but this tends to be from a developmental psychology perspective. Mukherjee (2020) has categorized children’s leisure into three types: structured, family, and casual leisure. Structured leisure is that which may or may not cost and involve some kind of scaffolding in terms of activities. School holiday programmes would tend to come under this category. Leisure is conceptualized as skills development. Parents are encouraged to invest time and resources in organized leisure to improve their children’s life chances, with families with higher incomes more likely to spend more to do this (Schneider, Hastings, and LaBriola 2018). Family leisure is that which CYP participate in as a family and would generally be organised by an adult when school is out. Casual leisure is not structured and is likely to be a site for CYP to have greater freedom to decide what they are doing and in what way. Casual leisure is problematized within public health approaches to school holidays, with a push for CYP to be engaged in structured leisure or more schooling. Nevertheless, we have only limited understanding of how CYP experience casual leisure during school holidays. A challenge for public health is also that casual leisure is less readily measurable than other types. Each of these leisure types must be considered together as inevitably there is a displacement effect. When children spend more time engaged in one form, they spend less time in another. We must consider the extent to which displacement of casual and family leisure with structured leisure is always positive or might generate the potential for negative unintended consequences.

Whilst casual leisure among school children is under-explored, there has been greater focus on leisure experiences of older adolescents and young adults. This could be because these young people are on the cusp of ‘being’ compared with younger children. Work carried out by Batchelor and colleagues (Batchelor et al. 2017; Batchelor et al. 2020) has highlighted the ways in which casual (or ‘pure’) leisure has been devalued and marginalized. Young people are subject to surveillance in public spaces, forcing them either into commercial leisure spheres that are future-orientated and cost money, or into private spheres or online. Similar issues have been identified by Bradford and McNamara (2015) when considering children’s leisure and health. They argue:

Undoubtedly, leisure spaces exist in which young people can and should engage in critical dialogue about health and well-being, perhaps as part of a wider discussion of citizenship, with sympathetic professionals. In other material and virtual settings, young people should be left to their own devices away from the perpetual adult gaze. (184)

They highlight the value in CYP having the opportunity to develop without the interference of adults. This is especially necessary for working-class young people, who have typically been the focus of concerns when they are beyond state control.

## Recommendations

This paper has highlighted some of the complexities involved in investigating children’s leisure during school holidays, and more specifically, their experiences of school holiday programmes. We have argued that CYP’s experiences of school holidays need to be studied. This includes all types of leisure experiences: structured, family and casual, without value judgements as to which is the preferred, or most potentially health enhancing. This understanding can be gained through cross-sectional or longitudinal surveys to investigate whether CYP with different socioeconomic characteristics are more or less likely to engage in different forms of leisure. More in-depth understanding of the ways in which CYP spend time out of school during holidays, and the value they place on the activities they engage in, can be gained through qualitative interviewing, either one-on-one, in pairs or in focus groups, ethnographic observation work, or through diaries/timeline or other creative approaches. These approaches can be carried out with CYP and/or carers, and potentially with other adults involved in supporting their health and wellbeing.

More specifically on holiday programmes, there is significant scope for CYP’s experiences of attending programmes to be examined further using similar methods to those above. There are also opportunities for CYP to be involved in co-designing their provision as active citizens. In addition, programme staff can provide interesting perspectives on their experiences of working with CYP in these environments, and parents can reflect on their families’ experiences of being supported through programmes. Whilst much of this work lends itself to a public health process evaluation approach, there is considerable scope for understanding the power dynamics among all relevant actors associated with programmes.

Interventions within public health research are typically evaluated through experimental or quasi-experimental designs. As part of these studies, researchers consider the programme theory and/or logic model that guides any programme. This provides an opportunity to reflect critically on the policy aims and organizational structures of programmes, such health and wellbeing, investment, childcare or poverty alleviation. Programme theories and logic models detail contextual information as well as inputs, outputs, and a range of outcomes (short, medium and long term). Care must be taken to avoid a narrow view of relevant outcomes. If we focus only on those that are more easily measured, such as physical activity, then we may miss the potential effectiveness of programmes to improve social and cultural aspects related to health, such as social capital, connectedness and lived citizenship.

## Conclusion

This paper has outlined the ways in which children’s right to leisure has been both championed and eclipsed when it is juxtaposed with children’s right to health. Through focusing on the ways in which CYP spend time during school holidays it has considered how the connection between leisure and health can be a means through which unequal leisure experiences can be revealed. CYP from families with the highest socioeconomic positions have the potential double advantage of structured leisure enhancing health and wellbeing, and providing opportunities for enjoyment and participation. For CYP from families with lower socioeconomic positions, opportunities to participate often relies on political will and state funding. In addition, lack of representation and co-design in structured leisure participation of CYP from families with lower socioeconomic positions makes it less likely that school holiday programmes are sites through which CYP’s citizenship can be realised, and more likely sites where structural inequalities are reproduced. This paper has called for further sociological research to understand CYP’s agency and participation in state provided leisure during school holidays. It has also considered the ways in which the narrowly defined focus on educational or physical health outcomes that currently dominates the discourse of CYP’s holiday programmes risks overshadowing social and emotional benefits of engagement in leisure. The role of casual leisure during school holidays in realizing CYP’s rights must also be explored, including the extent to which this is valued by CYP and the role casual leisure plays in CYP’s experiences of being, rather than as future-focussed ‘becomings’. Without this more holistic understanding, we are at risk of only a partial recognition of CYP’s experiences and practices, and a failure to uphold both their right to health and leisure.
